# Supplementary material for: Polyketide synthase gene clusters in the ericoid mycorrhizal fungus Oidiodendron maius: genome-wide prediction, expression profiling, and functional characterization of knockout mutants
Source: Front Plant Sci. 2026 Sep 16;17:1924430. doi: 10.3389/fpls.2026.1924430 (PMC13623903; doi:10.3389/fpls.2026.1924430)
Supplement: Supplementary file 1 [file SupplementaryFile1.zip › Supplementary Materials/Supplementary Folder 1.zip!/Oidma1_AssemblyScaffolds_Repeatmasked/knownclusterblast/cluster5/jgi.p_Oidma1_99913_mibig_hits.html]

| MIBiG Protein | Description | MIBiG Cluster | MiBiG Product | % ID | % Coverage | BLAST Score | E-value |
| --- | --- | --- | --- | --- | --- | --- | --- |
| CBF87076.1 | conserved\_hypothetical\_protein | BGC0001290 | Emericellamide\_A\_/\_emericellamide\_B\_biosynthetic\_gene\_cluster | 42 | 52.9085872576 | 476 | 7.5e-134 |
| EPE34349.1 | Acetyl-CoA\_synthetase-like\_protein | BGC0001035 | Pneumocandin\_biosynthetic\_gene\_cluster | 37 | 52.9085872576 | 404 | 2.8e-112 |
| AFT91380.1 | EcdI | BGC0000338 | Echinocandin\_B\_biosynthetic\_gene\_cluster | 39 | 52.9085872576 | 403 | 6.2e-112 |
| AMM63166.1 | AniI | BGC0001371 | Echinocandin\_B\_biosynthetic\_gene\_cluster | 39 | 52.9085872576 | 401 | 4e-111 |
| OAQ83766.1 | phenylacetyl-\_ligase\_protein | BGC0001358 | Leucinostatins\_biosynthetic\_gene\_cluster | 44 | 36.7497691597 | 316 | 1.7e-85 |
| BAO73255.1 | AMP\_dependent\_ligase/synthetase | BGC0000456\_c4 | Verlamelin\_biosynthetic\_gene\_cluster | 33 | 53.7396121884 | 315 | 2.9e-85 |
| BAH83502.1 | acyl-CoA\_synthetase | BGC0001253 | ACT-Toxin\_I\_biosynthetic\_gene\_cluster | 34 | 44.783010157 | 207 | 1.1e-52 |
| EHA28233.1 | hypothetical\_protein | BGC0001143 | Azanigerone\_biosynthetic\_gene\_cluster | 32 | 45.4293628809 | 189 | 1.8e-47 |
| AGY30675.1 | Ann3 | BGC0001298 | 4-Z-annimycin\_biosynthetic\_gene\_cluster | 29 | 45.6140350877 | 161 | 4.1e-39 |
| CAE14427.1 | long-chain-fatty-acid--CoA\_ligase\_(long-chain\_acyl-CoA\_synthetase) | BGC0001180\_c2 | Isopropylstilbene\_biosynthetic\_gene\_cluster | 27 | 54.2936288089 | 158 | 3.5e-38 |
| ABJ90146.1 | MoeA4 | BGC0000805 | Moenomycin\_biosynthetic\_gene\_cluster | 28 | 44.783010157 | 150 | 1.2e-35 |
| ADC79613.1 | BafX | BGC0000028 | Bafilomycin\_biosynthetic\_gene\_cluster | 26 | 44.1366574331 | 148 | 4.7e-35 |
| CAH10130.1 | feruloyl-CoA\_synthetase | BGC0000268 | Sch47554\_/\_Sch47555\_biosynthetic\_gene\_cluster | 26 | 45.2446906741 | 143 | 1.2e-33 |
| ABS74201.1 | YngI | BGC0001095 | Fengycin\_biosynthetic\_gene\_cluster | 25 | 50.6001846722 | 141 | 7.5e-33 |
| ADI58647.1 | AsuD3 | BGC0000187 | Asukamycin\_biosynthetic\_gene\_cluster | 27 | 44.5060018467 | 139 | 1.7e-32 |
| AEI98652.1 | CtcI | BGC0000209 | Chlortetracycline\_biosynthetic\_gene\_cluster | 27 | 34.1643582641 | 137 | 8.3e-32 |
| CAE17553.1 | acyl\_CoA\_ligase | BGC0000210 | Chromomycin\_A3\_biosynthetic\_gene\_cluster | 28 | 34.1643582641 | 137 | 1.1e-31 |
| ADE34493.1 | SsfL2 | BGC0000269 | SF2575\_biosynthetic\_gene\_cluster | 26 | 45.2446906741 | 130 | 1.3e-29 |
| AAF08801.1 | YngI | BGC0001103 | Mycosubtilin\_biosynthetic\_gene\_cluster | 25 | 50.8771929825 | 129 | 3e-29 |
| AFU65902.1 | DacH | BGC0000216 | Dactylocycline\_biosynthetic\_gene\_cluster | 27 | 34.7183748846 | 127 | 8.6e-29 |
| AEW31026.1 | short-chain\_acyl-CoA\_synthetase | BGC0000407 | Plipastatin\_biosynthetic\_gene\_cluster | 25 | 46.9067405355 | 126 | 2.5e-28 |
| AMY15066.1 | acyl-CoA\_synthetase/ligase | BGC0001339 | Squalestatin\_S1\_biosynthetic\_gene\_cluster | 24 | 51.2465373961 | 123 | 1.6e-27 |
| CAO85890.1 | aryl-coA\_ligase | BGC0000110 | Neoaureothin\_biosynthetic\_gene\_cluster | 26 | 45.4293628809 | 121 | 4.7e-27 |
| CAP20363.1 | equibactin\_siderophore\_biosynthetic\_protein | BGC0000347 | Equibactin\_biosynthetic\_gene\_cluster | 27 | 34.3490304709 | 119 | 3.1e-26 |
| ABC34346.1 | long-chain-fatty-acid--CoA\_ligase,\_putative | BGC0001102 | Malleilactone\_biosynthetic\_gene\_cluster | 24 | 46.2603878116 | 118 | 6.8e-26 |
| AHD25943.1 | ChdL | BGC0000208 | Chelocardin\_biosynthetic\_gene\_cluster | 27 | 36.3804247461 | 116 | 1.5e-25 |
| CAE02600.1 | putative\_acyl-coA\_ligase | BGC0000024 | Aureothin\_biosynthetic\_gene\_cluster | 26 | 44.3213296399 | 115 | 4.4e-25 |
| CAA07759.1 | acyl\_CoA\_ligase | BGC0000246 | Mithramycin\_biosynthetic\_gene\_cluster | 27 | 33.7950138504 | 113 | 1.7e-24 |
| CAK50779.1 | acyl\_CoA\_ligase | BGC0000247 | Mithramycin\_biosynthetic\_gene\_cluster | 27 | 33.7950138504 | 113 | 1.7e-24 |
| AFB35629.1 | EsmD2 | BGC0000935 | Esmeraldin\_biosynthetic\_gene\_cluster | 28 | 34.7183748846 | 102 | 2.3e-21 |
| CAJ96471.1 | Non-ribosomal\_peptide\_synthetase | BGC0000330 | Cupriachelin\_biosynthetic\_gene\_cluster | 25 | 38.7811634349 | 101 | 8.6e-21 |
| BAH33406.1 | 2,3-dihydroxybenzoate-AMP\_ligase | BGC0000371 | Heterobactin\_biosynthetic\_gene\_cluster | 27 | 34.0720221607 | 100 | 1.1e-20 |
| ADQ55476.1 | non-ribosomal\_peptide\_synthase\_dimodule | BGC0000350 | ET-743\_biosynthetic\_gene\_cluster | 25 | 38.5041551247 | 99 | 1.9e-20 |
| ALE27504.1 | 3-HPA:AMP\_ligase | BGC0001292 | A33853\_biosynthetic\_gene\_cluster | 26 | 34.1643582641 | 99 | 2.5e-20 |
| AAO62588.1 | peptide\_sythetase | BGC0001016 | Microcystin\_biosynthetic\_gene\_cluster | 22 | 50.5078485688 | 97 | 7.3e-20 |
| AMEG\_HIPS\_SALB\_00478 | Hydroxyl\_acids\_CoA\_ligase | BGC0001150 | Pamamycin\_biosynthetic\_gene\_cluster | 24 | 45.2446906741 | 97 | 7.3e-20 |
| AEI58866.1 | peptide\_synthetase | BGC0000455 | Vancomycin\_biosynthetic\_gene\_cluster | 24 | 50.323176362 | 97 | 1.2e-19 |
| ADZ24989.1 | proline\_adenylation\_protein | BGC0000380 | Leupyrrin\_biosynthetic\_gene\_cluster | 26 | 35.1800554017 | 96 | 2.1e-19 |
| BAH43766.1 | tyrocidine\_synthetase\_III | BGC0000452 | Tyrocidine\_biosynthetic\_gene\_cluster | 23 | 51.9852262235 | 96 | 2.1e-19 |
| ABQ96384.2 | fusaricidin\_synthetase | BGC0001152 | Fusaricidin\_biosynthetic\_gene\_cluster | 26 | 35.4570637119 | 94 | 6.2e-19 |
| BAH22764.1 | nonribosomal\_peptide\_synthetase | BGC0001018 | Micropeptin\_biosynthetic\_gene\_cluster | 24 | 50.784856879 | 94 | 8.1e-19 |
| BAH43870.1 | putative\_linear\_pentadecapeptide\_gramicidin\_synthetase\_LgrB | BGC0000367 | Gramicidin\_biosynthetic\_gene\_cluster | 25 | 34.810710988 | 94 | 8.1e-19 |
| AAL15617.1 | acetyl-coenzyme\_A\_synthetase | BGC0000270 | Simocyclinone\_biosynthetic\_gene\_cluster | 28 | 26.7774699908 | 92 | 2.3e-18 |
| AKA59431.1 | benzoate-CoA\_ligase | BGC0001202 | Landepoxcin\_biosynthetic\_gene\_cluster | 26 | 33.9796860572 | 92 | 3.1e-18 |
| AHD05621.1 | non-ribosomal\_peptide\_ligase\_domain\_protein | BGC0001033 | Paenilamicin\_biosynthetic\_gene\_cluster | 22 | 54.0166204986 | 91 | 6.8e-18 |
| AGM16413.1 | paenibacterin\_synthetase\_B | BGC0000400 | Paenibacterin\_biosynthetic\_gene\_cluster | 24 | 35.6417359187 | 91 | 8.9e-18 |
| AGM16414.1 | paenibacterin\_synthetase\_C | BGC0000400 | Paenibacterin\_biosynthetic\_gene\_cluster | 22 | 45.7987072946 | 91 | 8.9e-18 |
| ABC87508.1 | NRPS\_for\_pipecolate\_incorporation | BGC0001011 | Meridamycin\_biosynthetic\_gene\_cluster | 23 | 35.0877192982 | 89 | 2.6e-17 |
| CAA11795.1 | PCZA363.4 | BGC0000322 | Chloroeremomycin\_biosynthetic\_gene\_cluster | 24 | 53.4626038781 | 89 | 2.6e-17 |
| ctg1\_orf19 |  | BGC0001013 | Meridamycin\_biosynthetic\_gene\_cluster | 23 | 35.0877192982 | 89 | 2.6e-17 |
| AAX31555.1 | acyl-CoA\_ligase | BGC0000336 | Daptomycin\_biosynthetic\_gene\_cluster | 25 | 40.1662049861 | 89 | 3.4e-17 |
| AGM16412.1 | paenibacterin\_synthetase\_A | BGC0000400 | Paenibacterin\_biosynthetic\_gene\_cluster | 24 | 35.0877192982 | 89 | 3.4e-17 |
| AAZ03550.1 | McnA | BGC0000332 | Cyanopeptolin\_biosynthetic\_gene\_cluster | 23 | 49.3074792244 | 88 | 4.4e-17 |
| AIG79241.1 | Hypothetical\_protein | BGC0000419 | Ristomycin\_A\_biosynthetic\_gene\_cluster | 25 | 36.1034164358 | 88 | 4.4e-17 |
| AIW82280.1 | PuwC | BGC0001125 | Puwainaphycins\_biosynthetic\_gene\_cluster | 25 | 39.7045244691 | 87 | 7.5e-17 |
| AAX98210.1 | acyl\_CoA\_ligase | BGC0000052 | ECO-02301\_biosynthetic\_gene\_cluster | 24 | 31.9482917821 | 87 | 9.8e-17 |
| AED90002.1 | non-ribosomal\_peptide\_synthetase\_ThaA | BGC0000443 | Thanamycin\_biosynthetic\_gene\_cluster | 25 | 38.7811634349 | 87 | 9.8e-17 |
| CBJ79915.1 | putative\_Phenylalanine\_racemase\_(ATP-hydrolyzing) | BGC0001133 | Taxlllaid\_biosynthetic\_gene\_cluster | 21 | 50.1385041551 | 87 | 1.3e-16 |
| ABD14711.1 | CesA | BGC0000320 | Cereulide\_biosynthetic\_gene\_cluster | 22 | 48.6611265005 | 87 | 1.3e-16 |
| AEU11006.1 | NpnB | BGC0001029 | Nostophycin\_biosynthetic\_gene\_cluster | 22 | 46.999076639 | 87 | 1.3e-16 |
| CBJ79916.1 | putative\_Ornithine\_racemase | BGC0001133 | Taxlllaid\_biosynthetic\_gene\_cluster | 21 | 50.1385041551 | 87 | 1.3e-16 |
| ABD65957.1 | nonribosomal\_peptide\_synthetase | BGC0000341 | Enduracidin\_biosynthetic\_gene\_cluster | 26 | 37.02677747 | 86 | 1.7e-16 |
| AAY93355.1 | non-ribosomal\_peptide\_synthetase\_PvdJ | BGC0000413 | Pyoverdine\_biosynthetic\_gene\_cluster | 23 | 48.4764542936 | 86 | 1.7e-16 |
| BAH22765.1 | nonribosomal\_peptide\_synthetase | BGC0001018 | Micropeptin\_biosynthetic\_gene\_cluster | 23 | 52.6315789474 | 86 | 2.9e-16 |
| AAY93354.1 | non-ribosomal\_peptide\_synthetase\_PvdD | BGC0000413 | Pyoverdine\_biosynthetic\_gene\_cluster | 23 | 48.4764542936 | 86 | 2.9e-16 |
| AFV27434.1 | indigoidine\_synthase | BGC0000375 | Indigoidine\_biosynthetic\_gene\_cluster | 24 | 40.7202216066 | 85 | 3.7e-16 |
| ABJ97436.1 | MerP | BGC0001012 | Meridamycin\_biosynthetic\_gene\_cluster | 22 | 34.9953831948 | 85 | 3.7e-16 |
| ACY06285.1 | non-ribosomal\_peptide\_synthetase | BGC0001042 | Sanglifehrin\_A\_biosynthetic\_gene\_cluster | 25 | 38.5041551247 | 85 | 3.7e-16 |
| ABI26078.1 | OciB | BGC0000331 | Cyanopeptin\_biosynthetic\_gene\_cluster | 23 | 49.7691597415 | 85 | 4.9e-16 |
| EWS95124.1 | hypothetical\_protein | BGC0000306 | Arylomycin\_biosynthetic\_gene\_cluster | 25 | 29.7322253001 | 84 | 8.3e-16 |
| AIE77059.1 | peptide\_synthetase | BGC0000418 | Ristocetin\_biosynthetic\_gene\_cluster | 24 | 51.3388734995 | 84 | 8.3e-16 |
| ACK77757.1 | putative\_indigoidine\_synthase\_IndC | BGC0000727 | Auricin\_deoxysugar\_moieties\_biosynthetic\_gene\_cluster | 23 | 43.8596491228 | 84 | 8.3e-16 |
| AAN32979.1 | BarE | BGC0000962 | Barbamide\_biosynthetic\_gene\_cluster | 25 | 40.0738688827 | 84 | 1.1e-15 |
| CAB15186.3 | siderophore\_2,3-dihydroxybenzoate-glycine-threonine\_trimeric\_ester\_bacillibactin\_synthetase | BGC0000309 | Bacillibactin\_biosynthetic\_gene\_cluster | 26 | 35.3647276085 | 83 | 1.4e-15 |
| AKA59447.1 | non-ribosomal\_peptide\_synthetase | BGC0001203 | Clarexpoxcin\_biosynthetic\_gene\_cluster | 25 | 30.1939058172 | 83 | 1.4e-15 |
| CAC48361.1 | peptide\_synthetase | BGC0000311 | Balhimycin\_biosynthetic\_gene\_cluster | 25 | 50.5078485688 | 83 | 1.9e-15 |
| ABW00331.1 | amino\_acid\_adenylation\_domain | BGC0000333 | Cyclomarin\_biosynthetic\_gene\_cluster | 24 | 34.4413665743 | 82 | 2.4e-15 |
| ABD65958.1 | nonribosomal\_peptide\_synthetase | BGC0000341 | Enduracidin\_biosynthetic\_gene\_cluster | 26 | 28.9012003693 | 81 | 5.4e-15 |
| AJM89735.1 | PmxA | BGC0001192 | Colistin\_biosynthetic\_gene\_cluster | 24 | 41.2742382271 | 81 | 5.4e-15 |
| BAO84861.1 | putative\_non-ribosomal\_peptide\_synthetase | BGC0000414 | Quinocarcin\_biosynthetic\_gene\_cluster | 23 | 51.3388734995 | 81 | 7.1e-15 |
| AAO62585.1 | peptide\_sythetase\_polyketide\_synthase\_fusion\_protein | BGC0001016 | Microcystin\_biosynthetic\_gene\_cluster | 25 | 38.6888273315 | 79 | 2.7e-14 |
| AAZ03554.1 | McnE | BGC0000332 | Cyanopeptolin\_biosynthetic\_gene\_cluster | 23 | 52.4469067405 | 78 | 4.6e-14 |
| CBL93730.1 | NRPS | BGC0000360 | GE81112\_biosynthetic\_gene\_cluster | 25 | 44.2289935365 | 78 | 4.6e-14 |
| ALV86868.1 | Tlo22 | BGC0001406 | Telomycin\_biosynthetic\_gene\_cluster | 24 | 34.810710988 | 77 | 7.8e-14 |
| ABD65956.1 | nonribosomal\_peptide\_synthetase | BGC0000341 | Enduracidin\_biosynthetic\_gene\_cluster | 25 | 34.7183748846 | 77 | 7.8e-14 |
| AAU34202.1 | mannopeptimycin\_peptide\_synthetase\_MppA | BGC0000388 | Mannopeptimycin\_biosynthetic\_gene\_cluster | 23 | 35.0877192982 | 77 | 7.8e-14 |
| ADN13832.1 | Beta-ketoacyl\_synthase | BGC0001164 | 1-heptadecene\_biosynthetic\_gene\_cluster | 24 | 38.5964912281 | 77 | 1.3e-13 |
| CAE53352.1 | non-ribosomal\_peptide\_synthetase | BGC0000440 | Teicoplanin\_biosynthetic\_gene\_cluster | 23 | 39.8891966759 | 76 | 2.3e-13 |
| CAG15011.1 | peptide\_synthetase,\_module\_4-6 | BGC0000441 | Teicoplanin\_biosynthetic\_gene\_cluster | 23 | 39.8891966759 | 76 | 2.3e-13 |
| BAB69698.1 | iturin\_A\_synthetase\_A | BGC0001098 | Iturin\_biosynthetic\_gene\_cluster | 24 | 37.8578024007 | 76 | 2.3e-13 |
| AHD05678.1 | nonribosomal\_peptide\_ligase\_subunit | BGC0000402 | Paenilarvins\_biosynthetic\_gene\_cluster | 23 | 37.3037857802 | 76 | 2.3e-13 |
| AAN32981.1 | BarG | BGC0000962 | Barbamide\_biosynthetic\_gene\_cluster | 24 | 30.8402585411 | 76 | 3e-13 |
| ADH04679.1 | non-ribosomal\_peptide\_synthetase | BGC0001344 | Tubulysin\_biosynthetic\_gene\_cluster | 27 | 28.162511542 | 75 | 3.9e-13 |
| AJV88376.1 | MfnD | BGC0001214 | Marformycins\_biosynthetic\_gene\_cluster | 23 | 41.1819021237 | 75 | 3.9e-13 |
| ABD14712.1 | CesB | BGC0000320 | Cereulide\_biosynthetic\_gene\_cluster | 25 | 36.7497691597 | 75 | 5.1e-13 |
| CBD77754.1 | hypothetical\_protein | BGC0000974 | Crocacin\_biosynthetic\_gene\_cluster | 25 | 29.9168975069 | 75 | 5.1e-13 |
| AGE25479.1 | putative\_non-ribosomal\_peptide\_synthetase | BGC0001208\_c2 | Poaeamide\_biosynthetic\_gene\_cluster | 23 | 34.810710988 | 75 | 5.1e-13 |
| ABS74181.1 | BmyA | BGC0001090 | Bacillomycin\_biosynthetic\_gene\_cluster | 24 | 37.8578024007 | 74 | 6.6e-13 |
| CAF05648.1 | TubC\_protein | BGC0001053 | Tubulysin\_biosynthetic\_gene\_cluster | 23 | 39.1505078486 | 74 | 8.6e-13 |
| CBW45681.1 | putative\_amino\_acid\_adenyltransferase | BGC0000952\_c2 | Pristinamycin\_biosynthetic\_gene\_cluster | 24 | 30.1015697138 | 73 | 1.5e-12 |
| ADN26251.1 | ATP-dependent\_adenylase | BGC0000951 | Pacidamycin\_biosynthetic\_gene\_cluster | 24 | 37.3961218837 | 73 | 1.5e-12 |
| AAL33758.1 | putative\_non-ribosomal\_peptide\_synthetase | BGC0000421 | Safracin\_biosynthetic\_gene\_cluster | 22 | 35.8264081256 | 73 | 1.9e-12 |
| ABM21571.1 | CrpC | BGC0000975 | Cryptophycin\_biosynthetic\_gene\_cluster | 23 | 39.7968605725 | 72 | 2.5e-12 |
| KPN90369.1 | NunE | BGC0001416 | Nunapeptin\_/\_nunamycin\_biosynthetic\_gene\_cluster | 27 | 28.9935364728 | 72 | 3.3e-12 |
| ABX37385.1 | amino\_acid\_adenylation\_domain\_protein | BGC0000984 | Delftibactin\_biosynthetic\_gene\_cluster | 23 | 35.0877192982 | 72 | 3.3e-12 |
| CAD17793.1 | probable\_non\_ribosomal\_peptide\_synthetase\_protein | BGC0001363 | Ralsolamycin\_biosynthetic\_gene\_cluster | 25 | 37.2114496768 | 71 | 5.6e-12 |
| AAC83656.1 | dihydroaeruginoic\_acid\_synthetase | BGC0000412 | Pyochelin\_biosynthetic\_gene\_cluster | 24 | 31.3942751616 | 71 | 7.3e-12 |
| CAJ76292.1 | putative\_non-ribosomal\_peptide\_synthase | BGC0000972 | Colibactin\_biosynthetic\_gene\_cluster | 22 | 35.8264081256 | 71 | 7.3e-12 |
| AAM80536.1 | StaD | BGC0000290 | A47934\_biosynthetic\_gene\_cluster | 26 | 29.5475530933 | 71 | 9.5e-12 |
| CBW45640.1 | PII\_peptide\_synthetase | BGC0000952 | Pristinamycin\_biosynthetic\_gene\_cluster | 22 | 44.783010157 | 69 | 2.1e-11 |
| AID65225.1 | nonribosomal\_peptide\_synthetase | BGC0000335 | Cystomanamides\_biosynthetic\_gene\_cluster | 24 | 35.8264081256 | 69 | 2.1e-11 |
| ADD82940.1 | Bat2 | BGC0001099 | Kalimantacin\_/\_batumin\_biosynthetic\_gene\_cluster | 24 | 46.9067405355 | 69 | 2.1e-11 |
| AEF16021.1 | acyl-CoA\_synthase | BGC0000379 | Laspartomycin\_biosynthetic\_gene\_cluster | 23 | 42.1975992613 | 69 | 2.8e-11 |
| ABF88003.1 | putative\_long-chain-fatty-acid\_CoA\_ligase | BGC0000871 | VEPE\_/\_AEPE\_/\_TG-1\_biosynthetic\_gene\_cluster | 21 | 49.1228070175 | 69 | 2.8e-11 |
| AHD05679.1 | putative\_non-ribosomal\_peptide\_ligase/\_polyketide\_synthase\_hybrid | BGC0000402 | Paenilarvins\_biosynthetic\_gene\_cluster | 24 | 41.3665743306 | 67 | 1.1e-10 |
| BAH04162.1 | trsJ | BGC0000450 | Triostin\_A\_biosynthetic\_gene\_cluster | 24 | 34.5337026777 | 67 | 1.1e-10 |
| NP\_231840.1 | nonribosomal\_peptide\_synthetase\_VibF | BGC0000945\_c2 | Vibriobactin\_biosynthetic\_gene\_cluster | 23 | 47.8301015697 | 67 | 1.1e-10 |
| AAT70101.1 | CurF | BGC0001165 | Curacin\_biosynthetic\_gene\_cluster | 23 | 32.4099722992 | 67 | 1.4e-10 |
| BAE98155.1 | putative\_non-ribosomal\_peptide\_synthetase | BGC0000339 | Echinomycin\_biosynthetic\_gene\_cluster | 24 | 38.4118190212 | 67 | 1.4e-10 |
| AEE88284.1 | CurF | BGC0000976 | Curacin\_biosynthetic\_gene\_cluster | 23 | 32.4099722992 | 67 | 1.4e-10 |
| AAF99707.2 | syringopeptin\_synthetase | BGC0000438 | Syringopeptin\_biosynthetic\_gene\_cluster | 23 | 35.6417359187 | 66 | 2.3e-10 |
| AEC14347.1 | nonribosomal\_peptide\_synthetase | BGC0000377 | Koranimine\_biosynthetic\_gene\_cluster | 24 | 28.162511542 | 66 | 2.3e-10 |
| AET98906.1 | putative\_non-ribosomal\_peptide\_synthetase | BGC0000415 | Quinomycin\_biosynthetic\_gene\_cluster | 23 | 34.5337026777 | 66 | 2.3e-10 |
| AAX31558.1 | peptide\_synthetase\_2 | BGC0000336 | Daptomycin\_biosynthetic\_gene\_cluster | 25 | 40.9048938135 | 65 | 4e-10 |
| CCA89328.1 | mixed\_trans-AT\_type\_I\_polyketide\_synthase/nonribosomal\_peptide\_synthetase | BGC0001111 | Rhizopodins\_biosynthetic\_gene\_cluster | 24 | 28.9012003693 | 65 | 4e-10 |
| BAO73252.1 | non-ribosomal\_peptide\_synthetase | BGC0000456 | Verlamelin\_biosynthetic\_gene\_cluster | 23 | 36.4727608495 | 64 | 6.8e-10 |
| AHB82069.1 | non\_ribosomal\_peptide\_synthetase | BGC0001231 | Microsclerodermins\_biosynthetic\_gene\_cluster | 23 | 50.5078485688 | 62 | 3.4e-09 |
| ABG94125.1 | non-ribosomal\_peptide\_synthetase | BGC0000417 | Rhodochelin\_biosynthetic\_gene\_cluster | 24 | 34.4413665743 | 61 | 7.6e-09 |
| AEH59099.1 | amino\_acid\_adenylation\_domain-containing\_protein/NRPS | BGC0000385 | Lysobactin\_biosynthetic\_gene\_cluster | 23 | 44.783010157 | 61 | 9.9e-09 |
| ctg1\_orf6 |  | BGC0001109 | Pederin\_biosynthetic\_gene\_cluster | 25 | 25.1154201293 | 60 | 1.7e-08 |
| AAS47564.1 | mixed\_type\_I\_polyketide\_synthase/nonribosomal\_peptide\_synthetase | BGC0001108 | Pederin\_biosynthetic\_gene\_cluster | 25 | 25.1154201293 | 60 | 1.7e-08 |
| CAD29795.1 | peptide\_synthetase | BGC0001015 | Microcystin\_biosynthetic\_gene\_cluster | 22 | 38.8734995383 | 59 | 2.2e-08 |
| CCP45168.1 | Peptide\_synthetase\_MbtE\_(peptide\_synthase) | BGC0001021 | Mycobactin\_biosynthetic\_gene\_cluster | 25 | 39.6121883657 | 59 | 3.8e-08 |
| AAD44233.1 | PstA | BGC0000362 | Glycopeptidolipid\_biosynthetic\_gene\_cluster | 24 | 39.8891966759 | 57 | 1.4e-07 |
| AFJ23825.1 | WLIP\_synthetase\_B | BGC0000462\_c2 | WLIP\_biosynthetic\_gene\_cluster | 22 | 28.6241920591 | 57 | 1.4e-07 |
| AMB48444.1 | long-chain-fatty-acid--CoA\_ligase | BGC0001357 | Carbamidocyclophane\_biosynthetic\_gene\_cluster | 22 | 38.5041551247 | 56 | 3.2e-07 |
| AFH75322.1 | nonribosomal\_peptide\_synthetase | BGC0000425 | Sessilin\_biosynthetic\_gene\_cluster | 27 | 21.6066481994 | 56 | 3.2e-07 |
| CCM44336.1 | putative\_nonribosomal\_peptide\_synthetase | BGC0001056 | Zeamine\_biosynthetic\_gene\_cluster | 21 | 36.2880886427 | 54 | 7.1e-07 |
| AAD44234.1 | PstB | BGC0000362 | Glycopeptidolipid\_biosynthetic\_gene\_cluster | 24 | 45.1523545706 | 54 | 7.1e-07 |
| EAU38971.1 | hypothetical\_protein | BGC0001122 | Isoflavipucine\_biosynthetic\_gene\_cluster | 21 | 39.0581717452 | 52 | 2.7e-06 |
| AGD80622.1 | AMP-dependent\_synthetase\_and\_ligase | BGC0000394 | Naphthyridinomycin\_biosynthetic\_gene\_cluster | 23 | 28.9935364728 | 51 | 7.8e-06 |
